# Impact of Assisted Reproduction Techniques on Adverse Maternal Outcomes and on the Rate of Hospitalization in Maternal Intensive Care

**DOI:** 10.3390/medicina59112030

**Published:** 2023-11-17

**Authors:** Julie Collée, Laure Noel, Laurence Seidel, Frédéric Chantraine, Michelle Nisolle, Laurie Henry

**Affiliations:** 1 Obstetrics and Gynecology Department, Centre Hospitalier Universitaire de Liège, Citadelle Hospital, Boulevard du 12ème de Ligne 1, 4000 Liege, Belgiumlaurie.henry@chuliege.be (L.H.); 2 Center for Reproductive Medicine, University of Liège, Citadelle Hospital, Boulevard du 12ème de Ligne 1, 4000 Liege, Belgium; 3 Department of Statistical Analysis, University of Liège (ULiège), 4000 Liege, Belgium

**Keywords:** assisted reproductive treatment, obstetrical complications, maternal intensive care unit

## Abstract

*Background and Objective:* The aim of this retrospective cohort study is to evaluate the impact of assisted reproductive treatment (ART) on adverse maternal outcomes and the rate of hospitalization in maternal intensive care (MIC) in a tertiary university center in Liege, Belgium. *Materials and Methods:* This is a retrospective cohort study comparing two groups, 6557 patients who achieved pregnancy spontaneously and 330 patients who achieved pregnancy after ART, between January 2020 and December 2022. These patients were followed in the academic obstetrics department of Citadelle Hospital, Liège. The database of the ART center was compared with the database of the delivery unit to determine the cohort of patients who conceived after ART. Adverse maternal outcomes and MIC hospitalization rates were compared with between spontaneous pregnancies and ART groups. ART groups were also compared with each other. *Results:* The rate of hospitalization in maternal intensive care for patients who achieved pregnancy spontaneously was 12.1%, compared to 17.3% after ART. Comparing the rate of pre-eclampsia, 3.5% of spontaneous pregnancies were complicated by pre-eclampsia, while after ART, 10.9% of patients developed this complication during pregnancy. This rate was higher after IVF (12%) compared to intrauterine insemination and particularly after frozen embryo transfer (FET) in artificial cycle (17.9%). The birthweight of newborns after ART was also analyzed. A significant difference was obtained when comparing fresh embryo transfer with FET. *Conclusions:* Our study confirmed that FET in artificial cycle is a risk factor for pre-eclampsia and that fresh embryo transfer is associated with a higher rate of newborns with a lower percentile of birthweight. Our data showed that the rate of MIC hospitalization was significantly higher after ART but did not differ between groups.

## 1. Introduction

In recent decades, the use of assisted reproductive technology (ART) has increased worldwide [1]. This is particularly related to the easier use of gamete donation for homo- or monoparental projects and especially to the rising incidence of infertility. Indeed, it affects approximatively 12 percent of women of reproductive age. ART corresponds to all interventions that include the in vitro handling of both human oocytes and sperm or of embryos for the purpose of reproduction. In vitro fertilization (IVF) consists in an ovarian stimulation with gonadotrophins. There are two techniques for IVF: classic IVF and intracytoplasmic spermatozoid injection (ICSI) according to patient specificities such as sperm quality [2]. The number of frozen embryo transfers (FETs) especially increases and reaches around 40,000 FETs a year worldwide. This is related to the improvement of the cryopreservation procedure thanks to vitrification, elective single embryo transfer policy, “freeze-all” strategy and larger indications of pre-implantation genetic testing [3]. FET could be performed during artificial or natural cycles [2].

However, it is worth pointing out that, while its use continues to grow, ART is not without risk. Indeed, it is well known that pregnancies conceived after ART are associated with a higher risk of maternal and neonatal complications. Hypertensive disorders of pregnancies (HDPs) are more frequent after ART and especially after FET in artificial cycle [4] with a rate of 12.8%. HDPs include gestational hypertension and pre-eclampsia. Gestational hypertension is defined by hypertension (above 140/90 mmHg at rest) after 20 weeks’ gestation [5]. Pre-eclampsia is a specific disease during pregnancy and associates gestational hypertension with abnormal proteinuria and/or other findings such as signs of acute renal failure, hepatic dysfunction, neurological signs, hemolysis or thrombocytopenia. Neonatally, pre-eclampsia can lead to intrauterine growth retardation and iatrogenic prematurity, with delivery as the only known curative treatment for the mother [6]. The precise etiology of this condition is not yet known, but it is often associated with abnormal placentation. The syndrome of pre-eclampsia is heterogeneous, and its etiology differs according to whether it is early pre-eclampsia (before 34 days’ amenorrhea) or late pre-eclampsia (after 34 days’ amenorrhea). Early pre-eclampsia appears to be linked to abnormal trophoblast invasion and poor placental development [7]. Late pre-eclampsia, on the other hand, seems to be more frequently associated with constitutional factors, maternal lifestyle and poor systemic cardiovascular adaptation of the mother to pregnancy (BMI over 30, increased gestational weight gain, metabolic syndrome and maternal age over 35). Relaxin is a hormone secreted by the corpus luteum and is involved in the creation of the low-resistance vascular system. It helps maintain normal blood pressure and vascular compliance during pregnancy. Recent studies highlight the impact of relaxin deficiency in the pathogenesis of pre-eclampsia [8]. More specifically, low relaxin levels may be linked to late-onset pre-eclampsia. Therefore, FET in artificial cycle is principally associated with late-onset pre-eclampsia [9].

A meta-analysis of 12 studies published in 2021 enhances the higher rate of placenta accreta spectrum following ART and FET in artificial cycle [10]. Gestational diabetes is also higher in the population of ART with an RR of 1.53. Pregnancies after IVF and fresh embryo transfer have a higher rate of gestational diabetes than FET and ICSI [11]. Regarding the birthweight of neonates after ART, FET is related to neonates larger for the gestational age and fresh embryo transfer to neonates small for gestational age [12,13]. Patients can be hospitalized for those obstetrical complications [14].

Moreover, neonatal complications exist. The risk of stillbirth is increased after IVF/ICSI, with an odds ratio of 1.82 [15]. Congenital heart defects and urogenital tract malformations are also higher after ART, with no distinction in IVF or ICSI [16,17]. Urogenital tract malformations are especially hypospadias and cryptorchidism and are more frequent with multiple pregnancies than with singleton.

The aim of this retrospective cohort study is to compare the rate of obstetrical complications and of hospitalization in maternal intensive care between spontaneous pregnancies and pregnancies after ART and, which is far less studied in the literature, between the different types of ART.

## 2. Materials and Methods

### 2.1. Patients

This is a retrospective cohort study accepted by the Citadelle Hospital ethical committee on the 16 December 2022 (B4122022000032). Two groups were analyzed, spontaneous pregnancies and ART pregnancies. The patients were followed up in the academic obstetrics department at the Citadelle Hospital, Liège, from January 2020 to December 2022. Spontaneous pregnancies represent 6628 patients, and 343 patients conceived pregnancies after ART. Multiple pregnancies were excluded, resulting in 6557 patients who conceived naturally and 330 patients who conceived through ART. Among these 330 patients, there were 63 pregnancies obtained by intrauterine insemination (IUI) and 267 after in vitro fertilization (IVF): 117 after fresh embryo transfer and 150 after frozen embryo transfer.

The database of the ART center was compared with that of the delivery unit to determine the cohort of patients who had delivered a pregnancy resulting from ART.

### 2.2. Procedures

The choice of ART was determined by the physicians considering the age, the type and the duration of infertility. Patients who underwent IUI were patients with monoparental project, with ovulatory dysfunction, homosexual couples and without tubal infertility. Patients who underwent IVF were couples who failed in IUI, with tubal infertility, with vitrified oocytes, with a poor sperm quality or with older age. The selection of the preparation methods before FET was based on ovulatory status, previous FET attempts or physician preferences. The number and stage of embryos transferred depends on patients’ prognosis and embryo quality.

In the IUI group, patients were in spontaneous cycles or with a minor ovarian stimulation (clomifene citrate, aromatase inhibitors or low dose of gonadotropins). When one or two follicles measured 16 mm or more, ovulation was triggered by hCG, and the IUI was programmed the day after.

In the fresh embryo transfer group, one or two embryos were transferred on day 3 or on day 5 after retrieval of the oocyte. From the day after the ovarian pick-up, luteal phase was supported by the administration of oral dydrogesterone (Duphaston, Abbot, OLST, Netherlands). If the patient was pregnant, this treatment was continued until 8 weeks of gestation.

If the patient benefitted from a frozen embryo transfer in natural cycle, ultrasound and biological monitoring began on day 12 of the menstrual cycle and were repeated until the day of the ovulation trigger or the spontaneous LH surge. FET day was determined according to the day of ovulation, and up to two embryos were transferred depending on the stage of development (day 3 or day 5), embryo quality and patient history. Luteal phase was supported by vaginal progesterone (Utrogestan, Besins, France) and if the patient was pregnant until 8 weeks of gestation. For the group of FET in artificial cycle, transdermal estrogens were administrated by oral or transdermic administration. The treatment was started on the first day of the menstrual cycle. Vaginal progesterone (Utrogestan, Besins, France) was added when the endometrial thickness measured above 7 mm. FET was realized on the fourth or the fifth day after progesterone initiation. If pregnancy was achieved, estrogens and progesterone supplementation were continued until 12 weeks of gestation.

Obstetrical complications were obtained from the database of the delivery unit.

### 2.3. Outcomes

There is no standard definition of a maternal intensive care unit and the criteria to hospitalize patients in this type of unit differ between countries and units [18].

In this study, gestational hypertension is defined by hypertension (above 140/90 mmHg at rest) after 20 weeks’ gestation. Pre-eclampsia associates gestational hypertension with abnormal proteinuria and/or other maternal systemic failure.

Preterm delivery was defined as delivery before 37 weeks of gestation, early gestation before 28 weeks of gestation and late prematurity between 34 and 37 weeks of gestation [19]. Large for gestational age was defined as a birthweight above the 90th percentile, and small for gestational age below the 10th percentile. Intrauterine growth restriction was defined with a birthweight below the 3rd percentile or below the 10th percentile with a pulsatility index above the 95th percentile in the uterine artery [20]. Abnormal invasion and localization of the placenta were defined through the FIGO guidelines [21,22].

### 2.4. Statistical Analysis

Results were presented as means and standard deviations (SDs), quartiles (median, Q1 and Q3) and extremes (minimum and maximum) for quantitative variables, and as frequency tables for qualitative variables. For continuous variables, an ANOVA test was realized, and if there was a difference, a Scheffé test was performed for paired groups. To compare variables between groups, Student’s *t*-test was used for quantitative variables and Fisher’s exact test for qualitative variables. The association between two quantitative variables was measured using the correlation coefficient. Results are considered significant at the 5% uncertainty level (*p* < 0.05). Calculations were performed using SAS Entreprise version 9.4.

## 3. Results

### 3.1. Baseline Characteristics

**Figure 1:**
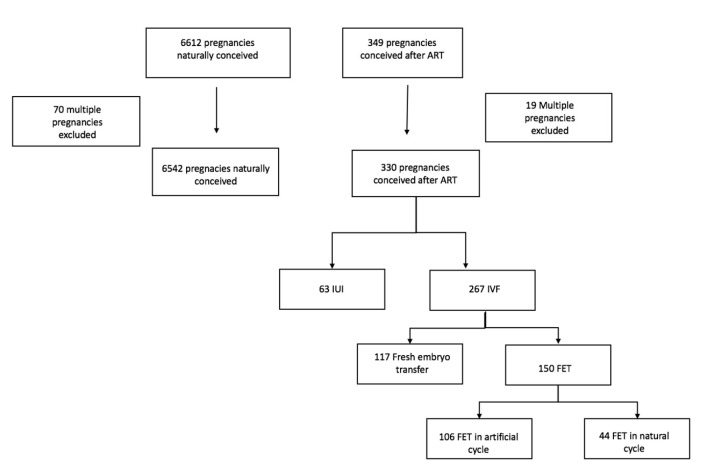
Flow diagram of patient screening and enrollment.

We screened 6557 pregnancies naturally conceived and 330 pregnancies conceived after assisted reproductive treatment. The 331 pregnancies conceived after ART were divided into 63 IUI and 267 IVF (Figure 1). Among the IUI group, 36 IUI were realized with the sperm of the partner (PS) and 27 with the sperm of a donor (DS). Among fresh embryo transfers, 99 were with PS and 18 from DS. In the FET group, 137 were realized with PS and 12 with DS. A list of the baseline characteristics of the patients included in the study is given in Table 1 and Table 2. Patients who became pregnant spontaneously were younger than in the ART group, especially in the IVF group. The difference between the age following natural conception and IUI was not significant. Patients in the spontaneous group had a lower BMI than patients in the IVF group and especially in FET in artificial cycle. Concerning ethnicity, the number of Caucasian patients was higher in the ART group with 77.6% of the patients compared to 61.3% in the spontaneous group. The percentage of patients from Africa and North Africa was higher in the spontaneous group (respectively, 19.7% vs. 11.8% and 14.5% vs. 7.6%). The tobacco consummation rate was the same in both groups. In the ART group, nulliparous patients were more frequent, whereas multiparous patients were higher in the spontaneous group.

### 3.2. Obstetrical Outcomes

Obstetrics outcomes are presented in Table 2.

#### 3.2.1. Hospitalization in Maternal Intensive Care

ART is associated with an increased risk of hospitalization in the maternal care unit. The reasons for hospitalization were pre-eclampsia (*n* = 20, 6.0%), abnormal insertion of the placenta (*n* = 10, 3.1%) and fetal death (*n* = 7, 2.3%).

In the subgroup analysis, IVF represented a higher risk of hospitalization in the maternal care unit compared to natural conception (17.3% vs. 12.1%, *p* = 0.002). Following IUI, the difference in hospitalization rate is not significant. Among IVF, the hospitalization rate was higher in the fresh embryo transfer group, 18.7%, compared to 12.1% in natural conception (*p* < 0.001).

#### 3.2.2. Hypertensive Disorders of Pregnancy

The risk of HDPs is higher for the pregnancies conceived after ART compared to natural conception (10.9% vs. 3.5%, respectively, *p* < 0.001). In the subgroup analysis, we compared IVF to natural conception. IVF represents the higher risk (12%), especially FET in artificial cycle (17.9% vs. 3.5%, *p* < 0.001). FET in natural cycle also has a higher risk of HDPs (9.1%) compared to fresh embryo transfer, in which HDPs will develop in 7.7% of the patients (*p* > 0.05).

Pre-eclampsia was diagnosed in 3% of the patients in natural conception and 6% after ART. FET in artificial cycle induced 7.5% of pre-eclampsia, and the difference between this group and natural conception was significant (*p* < 0.01). Within pre-eclampsia, patients who benefit from a pregnancy through ART had a higher risk of late-onset pre-eclampsia. The rate of early-onset pre-eclampsia did not differ between the groups.

Gestational hypertension represented 0.6% of the naturally conceived pregnancies in comparison with ART (5.1%, *p* < 0.001).

Early-onset pre-eclampsia did not differ between the ART group and the spontaneous group, but the difference was significant in late-onset pre-eclampsia (4.8% in the ART group and 2.1% in the spontaneous group, *p* = 0.003) and especially in FET in artificial cycle (6.6%, *p* = 0.008).

FET in artificial cycle influenced gestational hypertension, pre-eclampsia and especially late-onset pre-eclampsia.

#### 3.2.3. Preterm Birth

Considering hospitalization to prevent preterm birth and the use of tocolysis, ART and especially IVF had a higher rate, 8.7% and 9.2%, respectively, compared to natural conception, which represented 2.5% of the pregnancies (*p* < 0.001). However, preterm birth did not differ between the groups.

#### 3.2.4. Abnormal Invasion of the Placenta

The rate of abnormal invasion of the placenta including placenta praevia, vasa praevia and placenta accreta spectrum disorders differed between the groups. IVF had a higher rate of abnormal invasion of the placenta with 3.8% of the pregnancies compared to natural conception with 0.5% of the pregnancies (*p* < 0.001). This difference was significant in all the groups of IVF, including fresh embryo transfer, FET in natural cycle and FET in artificial cycle with, respectively, 2.6%, 4.5% and 4.8% of the pregnancies.

#### 3.2.5. Fetal Death

Fetal death was statistically more frequent in pregnancies conceived after ART (2.3%, *p* = 0.0001), both after IVF and IUI (respectively, 2% (*p* = 0.0014) and 3.3% (*p* = 0.015)), compared to natural conception (0.3%). In the IVF group, it is especially FET in artificial cycle which has a higher rate of fetal death (2.1%, *p* = 0.036).

#### 3.2.6. Birthweight

There was no difference concerning the birthweight between neonates conceived through ART and neonates conceived naturally. However, if we compared FET and fresh embryo transfer, the percentile of birthweight was lower in fresh embryo transfer (49.2 vs. 59.1, *p* = 0.0045). This difference was enhanced between fresh embryo transfer and FET in artificial cycle (49.2 vs. 60.4, *p* = 0.0034).

This diagram represents patients divided by groups and the exclusion of multiple pregnancies.

## 4. Discussion

The number of couples experiencing infertility is increasing, probably because of changes in lifestyle, the environment and the tendency to postpone childbearing. Since 2019, interest in the obstetrical complications of ART has been growing. In this retrospective cohort study, adverse maternal outcomes and characteristics of couples undergoing ART were analyzed. Although the sample size is small, there are trends and statistically significant results concerning the risk of obstetrical complications.

In our study, in in vitro fertilization, both frozen and fresh embryo transfers were performed in older patients than IUI, which confirmed published data [23].

The number of preterm births did not differ between the groups of patients undergoing ART compared to the literature [24]. Although hospitalizations to avoid preterm birth differed between natural and ART conceptions, there was no significant difference in real preterm birth. In the general population, the hospitalization rate was 12.9%, compared with 17.3% in pregnancies resulting from ART. MIC hospitalization was higher in the IVF group than in the IUI or than the spontaneous group. There are no data in the literature comparing those groups. The main reasons for MIC hospitalization were the development of pre-eclampsia, preterm birth, premature membrane rupture, intrauterine growth retardation and fetal death. This rate was relatively high but may be linked to the easy access to healthcare and hospitalization at our center. Additionally, the high level of stress of patients who underwent a pregnancy through ART could explain the rate of hospitalization in MIC, especially for risk of preterm birth [25].

The birthweight percentile was significantly higher in frozen than in fresh embryo transfer in our cohort. This had also been found in the literature [12]. The percentage of large for gestational age is higher after FET than fresh embryo transfer. In the literature, this difference is explained by the impact of the freezing medium with cryoprotectants used to limit the freezing damage. In fact, frozen embryos are preserved in a non-physiological storage fluid, and consequently, epigenetic changes in the embryo take place in order to withstand the hostile environment of the storage medium and freezing in liquid nitrogen [26]. In fresh embryo transfer, birthweight is more often below the third percentile [13], as confirmed by our data. The explanation suggested is the presence of a non-physiological environment with a high concentration of estrogens before the fresh embryo transfer. This environment could alter the placentation.

Abnormal invasion and abnormal localization of the placenta is also more frequent after ART and especially after IVF. Abnormal invasion of the placenta is often explained by age, multiparity and a history of cesarian delivery. In the literature, justifications proposed to this higher rate of abnormal placenta localization and development following IVF are more often older patients and those who are more likely to have a history of uterine surgery, including cesarean delivery or dilatation and curettage, compared with women who conceived with spontaneous conception [10].

Finally, our data suggested that pre-eclampsia is an obstetrical complication enhanced after ART, which also confirms the literature data. In our cohort, pre-eclampsia is higher in patients having undergone FET than fresh embryo transfer and especially in artificial cycle. Fresh embryo transfer does not enhance this rate. This phenomenon seems to be explained by the absence of the luteal body after FET in the artificial cycle. The corpus luteum secretes vasoactive substances such as VEGF and relaxin, which play a decisive role in placentation [27]. These substances enable the cardiovascular system to adapt and the spiral arteries to vasodilate, thus promoting optimal placentation. The absence of these vasoactive substances could explain the higher risk of pre-eclampsia in artificial cycles. Indeed, in these cycles, there is no ovulation, resulting in the absence of the corpus luteum. Artificial cycle could be used mostly for patients with ovulatory disorders, including mostly Type 3—premature ovarian insufficiency—and Type 4—polycystic ovarian syndrome—of the new HyPo-P classification of ovulatory disorders published last year by FIGO [28]. Late-onset pre-eclampsia is more frequent for those patients regarding the cardiovascular system maladaptation [9]. With the risk of pre-eclampsia estimated at 20% in pregnant patients after FET in artificial cycle, it seems important to warn our patients. Early detection of pre-eclampsia could be interesting for those patients. This detection results in a combined test including PlGF rate, maternal factors, mean blood pressure and pulsatility index of uterine arteries [29]. If a higher rate of pre-eclampsia is revealed, a treatment with acetylsalicylic acid is started in the end of the first trimester of pregnancy [30]. However, in case of FET, acetylsalicylic acid could be started earlier, during the first trimester. A prospective randomized controlled study could evaluate this treatment since the confirmation of intrauterine pregnancy.

This retrospective study is the first, to our knowledge, to compare the obstetrical impact of different types of ART and the rate of hospitalization in the maternal intensive care unit. Artificial inseminations are not often analyzed in studies of obstetrical complications. In fact, they are more often performed in patients with few fertility problems, less comorbidities and for whom infertility is more likely to be due to male factor, in homosexual couples or in single-motherhood projects. Nevertheless, IUI also increased the risk of obstetrical complication.

## 5. Conclusions

The aim of this retrospective study is to highlight and to raise awareness of obstetrical complications after ART, both after IVF and IUI. The rate of pre-eclampsia is increased in populations who have achieved pregnancy after FET, particularly in artificial cycles. This complication should be prevented, in particular by using the triple test for early detection of pre-eclampsia, or preventive treatment with aspirin from the beginning of pregnancy.

## Figures and Tables

**Table 1:** Baseline characteristics of the patients.

Variable		Spontaneous (a)	ART (b)	IUI (c)	IUI PS (d)	IUI SD (e)	IVF (FET and Fresh ET) (f)	Fresh ET (g)	FET Natural Cycle (h)	FET Artificial Cycle (i)	FET (j)
Number of patients		6542	330	63	36	27	267	117	44	106	150
Age (years) (SD)		30.96 ± 5.53	33.1 ± 4.45 (*p* < 0.0001)	31.5 ± 4.47 (*p* = 0.41)	31.1 ± 4.09 (*p* = 0.85)	32.1 ± 4.95 (*p* = 0.30)	33.5 ± 4.37 (*p* < 0.0001)	33.5 ± 4.45 (*p* < 0.0001)	32.8 ± 4.69 (*p* = 0.029)	33.7 ± 4.15 (*p* < 0.0001)	33.4 ± 4.32
BMI (kg/m^2^) (SD)		25.8 ± 5.43	25.1 ± 4.65 (*p* = 0.02)	25.6 ± 4.29 (*p* = 0.76)	26.0 ± 4.15 (*p* = 0.83)	25.0 ± 4.49 (*p* = 0.47)	24.9 ± 4.73 (*p* = 0.014)	25.3 ± 4.88 (*p* = 0.36)	25.4 ± 4.70 (*p* = 0.65)	24.3 ± 4.55 (*p* = 0.0079)	24.6 ± 4.59
Pregnancy range (%)	Nulliparous	2440 (38.1)	208 (63.1) (*p* < 0.0001)	46 (73) (*p* < 0.0001)	27 (75) (*p* < 0.0001)	19 (70.4) (*p* = 0.001)	162 (60.8) (*p* < 0.0001)	77 (65.8) (*p* < 0.0001)	25 (57.8) (*p* = 0.0085)	60 (56.6) (*p* = 0.0002)	85 (57) (*p* < 0.001)
	Multiparous	3961 (61.9)	122 (36.9)	17 (27)	9 (25)	8 (29.6)	105 (39.2)	40 (34.2)	19 (42.2)	46 (43.4)	65 (43)
Ethnicity (%)	Caucasian	4005 (61.3)	256 (77.6) (*p* < 0.0001)	58 (92.1) (*p* < 0.0001)	31 (86.1) (*p* = 0.043)	27 (100) (*p* = 0.0006)	198 (74.3) (*p* = 0.0005)	87 (74.4) (*p* = 0.066)	33 (75.6) (*p* = 0.12)	78 (73.6) (*p* = 0.17)	111 (74.2) (*p* < 0.01)
	Afro-American	951 (14.5)	25 (7.6)	3 (4.8)	3 (8.3)	0 (0)	22 (8.2)	9 (7.7)	2 (4.4)	11 (10.4)	13 (8.6) (*p* > 0.05)
	North African	1288 (19.7)	39 (11.8)	2 (3.2)	2 (5.6)	0 (0)	37 (13.8)	17 (14.5)	6 (13.3)	14 (13.2)	20 (13.2) (*p* > 0.05)
	Asian	279 (4.3)	10 (3)	0 (0)	0 (0)	0 (0)	10 (3.7)	4 (3.4)	3 (6.7)	3 (2.8)	6 (4.0) (*p* > 0.05)
	Others	14 (0.2)	0 (0)	0 (0)	0 (0)	0 (0)	0 (0)	0 (0)	0 (0)	0 (0)	0 (0) (*p* > 0.05)
Tobacco consumption (%)		734 (11.2)	38 (11.5) (*p* = 0.86)	10 (15.9) (*p* = 0.23)	4 (11.1) (*p* = 1)	6 (22.2) (*p* = 0.11)	28 (10.4) (*p* = 0.77)	14 (12) (0.77)	3 (6.7) (*p* = 0.48)	11 (10.4) (*p* = 0.88)	14 (9.3) (*p* > 0.05)

This table represents the baseline characteristics of the patients. Each group of ART pregnancies has been compared to spontaneous pregnancies. *p*-value is calculated with the comparison of ART pregnancies versus spontaneous pregnancies (a vs. b, a vs. c, a vs. d, a vs. e, a vs. f, a vs. g, a vs. h, a vs. i and a vs. j).

**Table 2:** Adverse maternal and perinatal outcomes.

Variable			Spontaneous (a)	ART (b)	IUI (c)	IUI PS (d)	IUI DS (e)	IVF (FET and Fresh Embryo Transfer) (f)	Fresh Embryo Transfer (g)	FET in Natural Cycle (h)	FET in Artificial Cycle (i)	FET (j)
Number of patients			6542	330	63	36	27	267	117	44	106	150
Weeks of gestation (SD)			38.9 ± 2.31	38.5 ± 3.56 (*p* = 0.0031)	38.8 ± 4.24 (*p* = 0.64)	38.7 ± 4.21 (*p* = 0.56)	38.9 ± 4.35 (*p* = 0.96)	38.5 ± 3.38 (*p* = 0.0018)	38.9 ± 2.42 (*p* = 0.92)	38.5 ± 2.74 (*p* = 0.066)	38.0 ± 4.36 (*p* = 0.0002)	38.2 ± 3.95
Birthweight (g) (SD)			3227 ± 618	3181 ± 661 (0.13)	3341 ± 399 (*p* = 0.15)	3315 ± 364 (*p* = 0.40)	3375 ± 446 (*p* = 0.22)	3145 ± 703 (*p* = 0.019)	3137 ± 573 (*p* = 0.12)	3155 ± 616 (*p* = 0.17)	3148 ± 856 (*p* = 0.2)	3150 ± 791
Percentile (%) (SD)			/	55.4 ± 27.3	57.9 ± 23.4	57.7 ± 18.8	58.3 ± 28.8	54.8 ± 28.1	49.2 ± 27.5	56.0 ± 27.0	60.4 ± 28.3	59.1 ± 27.9
MIC hospitalization (%)			790 (12.1)	57 (17.3) (*p* = 0.0046)	7 (11.1) (*p* = 1)	4 (11.1) (*p* = 1)	3 (11.1) (*p* = 1)	50 (18.7) (*p* = 0.0012)	22 (18.8) (*p* = 0.032)	8 (18.2) (*p* = 0.11)	20 (18.9) (*p* = 0.05)	28 (18.7) (*p* < 0.05)
HDP (%)	HDP		230 (3.5)	36 (10.9) (*p* < 0.0001)	4 (6.3) (*p* = 0.29)	2 (5.6) (*p* = 0.36)	2 (7.4) (*p* = 0.25)	32 (12.0) (*p* < 0.0001)	9 (7.7) (*p* = 0.038)	4 (9.1) (*p* = 0.021)	19 (17.9) (*p* < 0.0001)	23 (15.3) (*p* < 0.001)
	Pre-eclampsia	Pre-eclampsia	193 (3.0)	20 (6) (*p* = 0.0048)	2 (3.2) (*p* = 0.71)	1 (2.8) (*p* = 1)	1 (3.7) (*p* = 0.56)	18 (6.7) (*p* = 0.0018)	7 (6.0) (*p* = 0.089)	4 (9.1) (*p* = 0.15)	8 (7.5) (*p* = 0.015)	10 (7.3) (*p* < 0.02)
		Before 34 weeks	55 (0.8)	4 (1.2) (*p* = 0.53)	1 (1.6) (*p* = 0.42)	1 (2.8) (*p* = 0.27)	0 (0.0) (*p* = 1)	3 (1.1) (*p* = 0.50	1 (0.9) (*p* = 1)	0 (0.0) (*p* = 1)	1 (0.9) (*p* = 0.6)	1 (0.7) (*p* > 0.05)
		After 34 weeks	138 (2.1)	16 (4.8) (*p* = 0.0034)	1 (1.6) (*p* = 1)	0 (0.0) (*p* = 1)	1 (3.7) (*p* = 0.44)	15 (5.6) (*p* = 0.001)	6 (5.1) (*p* = 0.041)	2 (4.5) (*p* = 0.25)	7 (6.6) *p* = 0.0082)	9 (6) (*p* < 0.01)
	Gestational hypertension		37 (0.6)	17 (5.2) (*p* < 0.0001)	2 (3.2) (*p* = 0.053)	1 (2.8) (*p* = 0.19)	1 (3.7) (*p* = 0.15)	15 (5.6) (*p* < 0.0001)	2 (1.7) (*p* = 0.15)	2 (4.5) (*p* = 0.029)	11 (10.4) (*p* < 0.0001)	13 (8.7) (*p* < 0.001)
Abnormal invasion and location of the placenta (%)			33 (0.5)	10 (3.1) (*p* < 0.0001)	0 (0.0) (*p* = 1)	0 (0.0) (*p* = 1)	0 (0.0) (*p* = 1)	10 (3.8) (*p* < 0.0001)	3 (2.6) (*p* = 0.024)	2 (4.5) (*p* = 0.024)	5 (4.8) (*p* = 0.0003)	7 (4.7) (*p* < 0.001)
Hospitalization for risk of preterm birth (%)			166 (2.5)	27 (8.7) (*p* < 0.0001)	4 (6.5) (*p* = 0.075)	1 (2.8) (*p* = 0.60)	3 (11.5) (*p* = 0.028)	23 (9.2) (*p* < 0.0001)	9 (8.3) (*p* = 0.0022)	3 (7.3) (*p* = 0.092)	11 (11.0) (*p* < 0.0001)	14 (9.9) (*p* < 0.001)
Preterm birth (%)			731 (11.2)	39 (12.1) (*p* = 0.65)	2 (3.3) (*p* = 0.061)	1 (2.9) (*p* = 0.17)	1 (3.8) (*p* = 0.35)	37 (14.1) (*p* = 0.16)	13 (11.3) (*p* = 0.88)	8 (18.2) (0.16)	16 (15.5) (*p* = 0.16)	24 (16.3) (*p* > 0.05)
Fetal death (%)			19 (0.3)	7 (2.3) (*p* < 0.0001)	2 (3.3) (*p* = 0.015)	1 (2.9) (*p* = 0.099)	1 (3.8) (*p* = 0.076)	5 (2.0) (*p* = 0.0015)	2 (1.8) (*p* = 0.046)	1 (2.4) (*p* = 0.12)	2 (2.1) (*p* = 0.0036)	3 (2.2) (*p* < 0.01)
Birthweight <= P3 (%)			185 (2.8)	10 (3.1) (*p* = 0.73)	1 (1.6) (*p* = 1)	0 (0.0) (*p* = 0.63)	1 (3.8) (*p* = 0.53)	9 (3.4) (*p* = 0.57)	7 (6.1) (*p* = 0.05)	0 (0.0) (*p* = 0.64)	2 (1.9) (*p* = 1)	2 (1.4) (*p* > 0.05)
Birthweight <= P10 (%)			540 (8.3)	24 (7.4) (*p* = 0.76)	3 (4.9) (*p* = 0.48)	0 (0.0) (*p* = 0.11)	3 (11.5) (*p* = 0.47)	21 (8.0) (*p* = 1)	10 (8.7) (*p* = 0.86)	3 (6.8) (*p* = 0.79)	8 (7.8) (*p* = 1)	11 (7.5) (*p* > 0.05)
Birthweight >= P97 (%)			273 (4.2)	13 (4.0) (*p* = 1)	3 (4.9) (*p* = 0.74)	1 (2.9) (*p* = 0.1)	2 (7.7) (*p* = 0.30)	10 (3.8) (*p* = 0.88)	3 (2.6) (*p* = 0.63)	1 (2.3) (*p* = 1)	6 (5.8) (*p* = 0.45)	7 (4.8) (*p* > 0.05)
Birthweight >= P90 (%)			920 (14.2)	37 (11.5) (*p* = 0.19)	5 (8.2) (*p* = 0.26)	2 (5.7) (*p* = 0.22)	3 (11.5) (*p* = 1)	32 (12.2) (*p* = 0.42)	10 (8.7) (*p* = 0.10)	3 (6.8) (*p* = 0.2)	19 (18.4) (*p* = 0.25)	22 (15.0) (*p* > 0.05)

This table represents the adverse perinatal outcomes. Each group of ART pregnancies has been compared to spontaneous pregnancies. *p*-value is calculated with the comparison of ART pregnancies versus spontaneous pregnancies (a vs. b, a vs. c, a vs. d, a vs. e, a vs. f, a vs. g, a vs. h, a vs. i and a vs. j).

## Data Availability

Data available on request due to restrictions of privacy. The data presented in this study are available on request from the corresponding author.
